# Protein Trafficking or Cell Signaling: A Dilemma for the Adaptor Protein TOM1

**DOI:** 10.3389/fcell.2021.643769

**Published:** 2021-02-26

**Authors:** Tiffany G. Roach, Heljä K. M. Lång, Wen Xiong, Samppa J. Ryhänen, Daniel G. S. Capelluto

**Affiliations:** ^1^Protein Signaling Domains Laboratory, Department of Biological Sciences, Fralin Life Sciences Institute, and Center for Soft Matter and Biological Physics, Virginia Tech, Blacksburg, VA, United States; ^2^Division of Hematology, Oncology, and Stem Cell Transplantation, Children’s Hospital, and Pediatric Research Center, The New Children’s Hospital, University of Helsinki and Helsinki University Hospital, Helsinki, Finland; ^3^Department of Anatomy and Stem Cells and Metabolism Research Program, Faculty of Medicine, University of Helsinki, Helsinki, Finland

**Keywords:** TOM1, TOL, endosome, ESCRT, TOLLIP, Endofin, phosphoinositides

## Abstract

Lysosomal degradation of ubiquitinated transmembrane protein receptors (cargo) relies on the function of Endosomal Sorting Complex Required for Transport (ESCRT) protein complexes. The ESCRT machinery is comprised of five unique oligomeric complexes with distinct functions. Target of Myb1 (TOM1) is an ESCRT protein involved in the initial steps of endosomal cargo sorting. To exert its function, TOM1 associates with ubiquitin moieties on the cargo *via* its VHS and GAT domains. Several ESCRT proteins, including TOLLIP, Endofin, and Hrs, have been reported to form a complex with TOM1 at early endosomal membrane surfaces, which may potentiate the role of TOM1 in cargo sorting. More recently, it was found that TOM1 is involved in other physiological processes, including autophagy, immune responses, and neuroinflammation, which crosstalk with its endosomal cargo sorting function. Alteration of TOM1 function has emerged as a phosphoinositide-dependent survival mechanism for bacterial infections and cancer progression. Based on current knowledge of TOM1-dependent cellular processes, this review illustrates how TOM1 functions in coordination with an array of protein partners under physiological and pathological scenarios.

## Introduction

To attenuate signaling, cells are able to ubiquitinate and remove cell-surface membrane proteins by a concerted process involving internalization of the proteins by endocytosis, followed by delivery to the endolysosomal membrane system for proteolysis. At the endosomal compartments, the recognition of the ubiquitin moieties of transmembrane proteins (referred to as cargo) is driven by the action of protein complexes that include Endosomal Sorting Complexes Required for Transport (ESCRT) proteins. This machinery allows major changes in endosomal membrane shape that lead to membrane invagination and scission, generating cargo-containing intralumenal vesicles. Thus, cargoes are restricted to these subcompartments for an efficient proteolytic process. Some of the earliest cargo sorting ESCRT proteins are in the target of Myb1 (TOM1) protein family, which includes TOM1 and its close relatives TOM1-like 1 (TOM1-L1) and 2 (TOM1-L2). This review focuses on both well-known and emerging functions and regulation of TOM1 under physiological and pathological scenarios. Readers interested in the function and regulation of other ESCRT proteins are referred to recent review articles (Norris and Grant, 2020; Vietri et al., 2020).

## TOM1 and the Endosomal Ubiquitin-Dependent Sorting Pathway

When dispensable, membrane proteins are modified by ubiquitination, internalized, and transported to the endosomal surface where they can be either recycled to the plasma membrane via the endosomal recycling pathway or degraded in late endosomes or lysosomes in animals or vacuoles in plants. Ubiquitin can be covalently attached as a single unit (monoubiquitination), conjugated to multiple sites in a tagged protein (multi-monoubiquitination), or attached as a polyubiquitin chain. Polyubiquitin chains can adopt either packed (i.e., ubiquitin-Lys48 chains) or flexible (i.e., ubiquitin-Lys63 chains) conformations (reviewed in Scott et al., 2015). A tetraubiquitin chain, linked through Lys48, is apparently the minimal chain length required for efficient proteasomal targeting (Thrower et al., 2000), whereas Lys63-linked hexaubiquitin seems to be the minimal length for recognition by the ESCRT proteins Hrs and STAM1/2 (Nathan et al., 2013).

In both animals and plants, degradation of cargo relies on the assembly of the ESCRT proteins at the surface of early endosomes, but complex initiation might occur at the plasma membrane (Mayers et al., 2013). The ESCRT apparatus recognizes cargo conjugated with single ubiquitin or polyubiquitin chains, and its protein components sequentially transport cargo to intralumenal vesicles where cargo is loaded. The ESCRT apparatus includes four core complexes, ESCRT-0, ESCRT-I, ESCRT-II, and ESCRT-III, as well as supporting auxiliary proteins (Weeratunga et al., 2020). Each of the ESCRT-0, ESCRT-I, and ESCRT-II complexes is composed of proteins with ubiquitin-binding domains for cargo recognition and phosphatidylinositol 3-phosphate (PtdIns3P)-binding domains for endosomal membrane anchoring.

**FIGURE 1:**
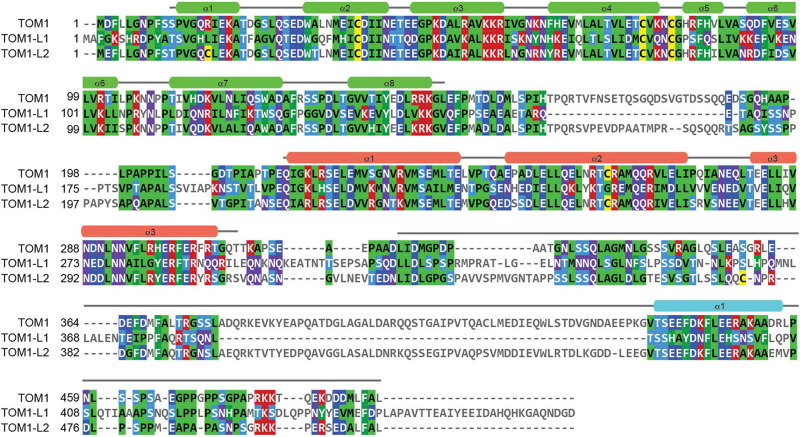
Sequence analysis of the TOM1 family of proteins. Amino acid sequences corresponding to Human TOM1 (accession O60784), TOM1-L1 (accession O75674), and TOM1-L2 (accession Q6ZVM7) were aligned using the Clustal Omega sequence alignment program and reformatted and colored using MView. The boundaries and secondary structure elements of the VHS, GAT, and C-terminal domains for TOM1 are shown in green, salmon, and cyan, respectively.

In mammals, the canonical ESCRT-0 complex is composed of Hrs and STAM1/2 proteins (Henne et al., 2011). Both Hrs and STAM1/2 bind cargo through their double-sided ubiquitin-interacting motif (UIM) and Vps27, Hrs, STAM (VHS) domains (Mizuno et al., 2003; Hirano et al., 2006; Ren and Hurley, 2010). Anchoring of canonical ESCRT-0 to endosomal membranes is through PtdIns3P recognition *via* the Fab1, YOTB, Vac1, EEA1 (FYVE) domain of Hrs (Raiborg et al., 2001). In addition to the canonical ESCRT-0 complexes, functionally related proteins, including TOM1, TOM1-L1, TOM1-L2, Toll-interacting protein (TOLLIP), and Endofin, have been proposed to work in parallel to ESCRT-0 as alternative ESCRT-0 early cargo transporters (Shields and Piper, 2011; Capelluto, 2012; Haglund and Dikic, 2012). The TOM1 protein family is modular with a VHS domain at the N-terminus, a central GAT domain, and a long, C-terminal domain (Figure 1). Phylogenetic analysis of the TOM1 family of proteins predicts close relationship among *Caenorhabditis elegans* TOM1, *Drosophila melanogaster* TOM1, and human TOM1, TOM1-L1, and TOM1-L2 proteins, but shows divergency for both plant TOM1-like (TOL) proteins and GGA proteins (Winter and Hauser, 2006). Thus, these findings suggest that TOM1, TOM1-L1, and TOM1-L2 may exhibit redundant functions. This observation can also be applied to plants, in which the TOM1 family of proteins is markedly expanded in *Arabidopsis thaliana* and *Oryza sativa*, with the presence of nine genes encoding VHS and GAT domain-containing proteins (Winter and Hauser, 2006).

The TOM1 family of proteins are ubiquitously present in human tissues. TOM1 displays enhanced mRNA expression in skeletal muscle, heart, placenta, and liver^1^ (Seroussi et al., 1999). TOM1-L2 shows a similar mRNA expression pattern but with enriched expression in the heart muscle (see Footnote 1). Interestingly, the expression of TOM1-L1 mRNA predominates in the gastrointestinal tract, with increased expression in the small intestine, as well as in liver and kidney (see Footnote 1). This variation in expression pattern is not surprising as TOM1-L1 is the most divergent of the TOM1 family of proteins (Figure 1).

As an ESCRT-0 component, TOM1 interacts with TOLLIP, Endofin, ubiquitin, and clathrin (Table 1; Yamakami et al., 2003; Katoh et al., 2004, 2006; Seet et al., 2004; Seet and Hong, 2005; Xiao et al., 2015) and transports cargo, as demonstrated by its role in trafficking the delta opioid receptor (Lobingier et al., 2017), interleukin 1-receptor, type 1 (IL-1R1) (Brissoni et al., 2006), and the cargo within mitochondrial-derived vesicles (Table 2; Ryan et al., 2020). TOM1 and TOM1-L1 also interface with the ESCRT-I tumor susceptibility gene 101 protein in *Dictyostelium* (Blanc et al., 2009) and humans (Puertollano, 2005), respectively (Table 2).

**TABLE 1:** Interactions of TOM1 family proteins.

Protein	Binding partner	References
TOM1	TOLLIP	Yamakami et al., 2003; Katoh et al., 2004, 2006; Xiao et al., 2015
	Clathrin heavy chain	Yamakami et al., 2003; Katoh et al., 2006
	Ubiquitin, Lys48-, Lys63-, Lys29-, and Lys33-linked polyubiquitin chains	Yamakami et al., 2003; Nathan et al., 2013; Zhao et al., 2017
	Endofin	Seet et al., 2004; Seet and Hong, 2005
	Hrs	Puertollano, 2005
	TSG101	Puertollano, 2005; Blanc et al., 2009
	Myosin VI	Tumbarello et al., 2012; Chen et al., 2019
	Phosphatidylinositol 5-phosphate	Boal et al., 2015; Xiong et al., 2019
TOM1-L1	Grb2*	Seykora et al., 2002; Liu et al., 2009
	Ubiquitin	Katoh et al., 2004
	TOLLIP	Katoh et al., 2004, 2006
	Hrs	Puertollano, 2005
	TSG10	Puertollano, 2005; Blanc et al., 2009
	Clathrin heavy chain	Katoh et al., 2006; Collin et al., 2007; Liu et al., 2009
	Src kinases	Collin et al., 2007
	EGFR*	Liu et al., 2009
	p85 subunit of PI3K*	Seykora et al., 2002
TOM1-L2	Clathrin heavy chain	Katoh et al., 2006
	TOLLIP	Katoh et al., 2006
	Myosin VI	Tumbarello et al., 2012; Zakrzewski et al., 2020
	Lys48- and Lys63-linked polyubiquitin chains	Nathan et al., 2013
TOL2/6	Lys63-linked polyubiquitin chains	Moulinier-Anzola et al., 2020
TOL6	ESCRT-I component VPS23A	Moulinier-Anzola et al., 2020

*These proteins require Tyr phosphorylation of TOM1-L1.

**TABLE 2:** Functions of TOM1 family proteins.

Protein	Function	References
TOM1	Recruitment of clathrin to endosomal membranes	Seet and Hong, 2005
	IL-1R1 trafficking	Brissoni et al., 2006
	Modulation of TLR2/4 recycling	Oglesby et al., 2010
	Amphisome formation	Tumbarello et al., 2012; Chen et al., 2019
	Delta opioid receptor trafficking	Lobingier et al., 2017
	Cargo trafficking in mitochondrial-derived vesicles	Ryan et al., 2020
TOM1-L1	Regulation of Src tyrosine kinase activity	Franco et al., 2006; Collin et al., 2007
	Endocytosis of EGFR	Liu et al., 2009
	Increasing invasiveness of ERBB2 breast cancer cells	Chevalier et al., 2016
TOM1-L2	Tumor suppression	Girirajan et al., 2008
	Acrosome formation	Zakrzewski et al., 2020
TOL1 to 9 proteins	Regulation of plant morphogenesis through PIN trafficking	Korbei et al., 2013
	Regulation of plant immune response	Conlan et al., 2018
	BOR1 protein trafficking	Yoshinari et al., 2018

**FIGURE 2:**
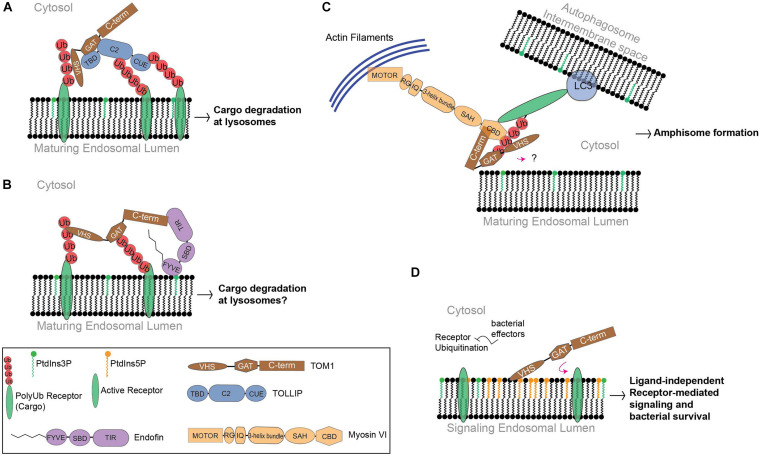
The function of TOM1 in mammals. **(A)** Cargo is delivered to early endosomes through vesicular transport. TOM1 is recruited by PtdIns3P-bound TOLLIP to these compartments, favoring cargo clustering at maturing endosomes. **(B)** Endofin recruits TOM1 to early endosomes. Although the function of this complex is unknown, it is possible that it transports cargo for degradation. **(C)** To mediate amphisome formation, Myosin VI is proposed to bridge endosomal and autophagosomal compartments in coordination with TOM1 and ubiquitinated receptors, which in turn, make contact with the microtubule-associated protein 1A/1B-light chain 3 (LC3) receptors at autophagosomal surfaces. In this proposed model, TOM1 can also make contact with polyubiquitin chains of receptors through VHS and GAT domains. The contact of TOM1 to endosomal surfaces is unknown. TIR, TOM1-interacting region; SBD, Smad-binding domain; RG, reverse gear region; IQ, isoleucine glutamine motif; SAH, single α-helix region; CBD, C-terminal globular cargo-binding domain. **(D)** Under bacterial infection conditions, PtdIns5P accumulates at signaling endosomes. Ubiquitination processes are subverted by bacterial effectors. PtdIns5P accumulation favors TOM1 sequestration, which promotes ligand-independent receptor-mediated signaling and cell survival.

TOM1 interacts with ubiquitin and polyubiquitin chains through its VHS and GAT domains (Figure 2A; Yamakami et al., 2003; Ren and Hurley, 2010). Unlike the observed selectivity of the canonical ESCRT-0 components for Lys63-linked polyubiquitin chains, TOM1, TOM1-L2, and TOLLIP bind Lys48- and Lys63-linked polyubiquitin topologies, with a slight preference for the latter (Table 1; Nathan et al., 2013; Lu et al., 2014). In addition, TOM1 has also been shown to interact with Lys29- and Lys33-linked polyubiquitin chains (Table 1; Zhao et al., 2017). It is currently unknown how TOM1 distinguishes between topologically distinct polyubiquitin chains, which represent functionally distinct intracellular signals.

**FIGURE 3:**
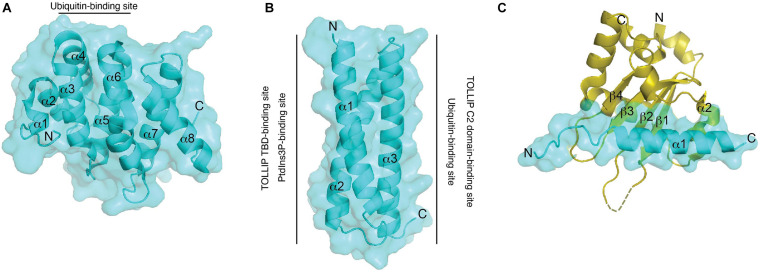
Structural features and binding properties of TOM1 domains. Surface and ribbon representations of the TOM1 VHS **(A)**, GAT **(B)**, and MBM bound to Myosin VI CBD **(C)**. Regions at which ligands bind to the TOM1 VHS and GAT domains are shown. An unresolved structural region in Myosin VI CBD is indicated as a dotted region.

One of the cargo recognition modules of TOM1 is the VHS domain. The VHS domain adopts a right-handed superhelix structure composed of eight α-helices (Figure 3A; Bonifacino, 2004). The residues located in α-helices α1–α5 and α7, but not those found in α6 and α8, are highly conserved among VHS domains (Kirchhausen, 2002). The divergency of α-helices 6 and 8 is supported by their arrangement in GGA proteins, in which they generate a groove that is required for specific interactions with acidic-cluster-dileucine sorting signals of transmembrane receptors, which are reported to be absent in other VHS domains (Misra et al., 2002; Shiba et al., 2002). Indeed, the Tepsin VHS domain lacks α-helix 8 and, consequently, is unable to recognize acidic-cluster-dileucine sorting signals, ubiquitin, and phosphoinositides (Archuleta et al., 2017). The structure of the TOM1 VHS domain shares several features with other VHS domains, but its α-helix 5 is shorter and its α-helix 8 orients away from the remaining α-helices of the protein (Figure 3A; Misra et al., 2000; Ellena et al., 2017).

Ubiquitin sorting signals are recognized by ubiquitin-binding domains, which transiently bind to a hydrophobic conserved surface patch in ubiquitin (Kliza and Husnjak, 2020). Binding of VHS domains to ubiquitin is mediated through residues located on α-helices 2 and 4 (Figure 3A), which contact a hydrophobic patch of ubiquitin made by residues Ile44, Gly47, and Val70 (Ren and Hurley, 2010). The affinity for ubiquitin interaction ranges in the high micromolar/low millimolar range, but it increases more than twofold by interactions with Lys48- and Lys63-linked tetraubiquitin chains (Ren and Hurley, 2010). Thus, the presence of several ubiquitin-binding domains in ESCRT proteins may contribute to an increase in the overall affinity for cargo for an efficient sorting process. TOM1, in coordination with TOLLIP, promotes the lysosomal degradation of IL-1R1 (Brissoni et al., 2006) and the cargo within mitochondrial-derived vesicles (Ryan et al., 2020; Table 2). TOLLIP is also modular with an N-terminal TOM1 binding domain (TBD) (Yamakami et al., 2003), a central PtdIns3P-binding conserved 2 (C2) domain (Ankem et al., 2011), and a C-terminal coupling of ubiquitin to endoplasmic reticulum degradation (CUE) domain (Azurmendi et al., 2010). Binding of TOM1 to TOLLIP occurs *via* a coupled folding and binding mechanism, in which the first two α-helices of the GAT domain fold TOLLIP TBD at its N-terminus, whereas the third α-helix of the GAT domain contacts the TOLLIP C2 domain (Figure 3B) and inhibits binding of TOLLIP to PtdIns3P (Xiao et al., 2015). Inhibition of TOLLIP’s PtdIns3P binding by TOM1 might be critical for engaging the ubiquitin-binding TOLLIP C2 domain to bind cargo (Mitra et al., 2013). Given the weak binding affinity of the ubiquitin-binding domains of TOM1 and TOLLIP for ubiquitin (Azurmendi et al., 2010; Mitra et al., 2013; Xiong et al., 2019), the simultaneous binding of ubiquitinated cargo enables increase of cargo clustering for more efficient degradation. Interestingly, the third α-helix of TOM1 GAT also associates to cargo through ubiquitin (Figure 3B) in the absence of TOLLIP (Xiao et al., 2015, 2016). Thus, TOM1 can use its GAT domain to contact cargo independently of TOLLIP; however, this can also occur in complex with other ESCRT-0 proteins such as Endofin and Hrs.

Unlike TOM1-L1, TOM1-L2, and Hrs, the carboxy−terminal domain of TOM1 (residues 300–492) specifically interacts with the carboxy−terminal region of Endofin (Table 1; Seet et al., 2004; Seet and Hong, 2005). Endofin is localized to early endosomes through the interaction of its FYVE domain with membrane-embedded PtdIns3P (Seet and Hong, 2001), recruiting TOM1 to these compartments (Figure 2B; Seet and Hong, 2005). Binding of TOM1 to Endofin also facilitates clathrin recruitment to endosomal membranes (Seet and Hong, 2005); however the precise function of clathrin in complex with TOM1 and Endofin at early endosomes is unknown. More recently, both TOM1 and Endofin have been shown to be targeted by the complement-derived anaphylatoxin C5a (Wood et al., 2020). Prior exposure with C5a reduces the phosphoprotein response of neutrophils against *Staphylococcus aureus* infection. TOM1 and Endofin display impaired phosphorylation in the presence of C5a, which is correlated with selective inhibition of the PtdIns3P producing enzyme VPS34 (Wood et al., 2020). As the V-type ATPase phosphorylation is also altered, exposure to C5a may affect the TOM1- and Endofin-dependent phagosomal maturation process in neutrophils.

## Plant TOM1-Like (TOL) Proteins, Orthologs of Mammalian TOM1 Proteins

The TOM1 subfamily exhibits more members in plants than animals, with nine TOM1-like proteins (TOL1-9) identified in *Arabidopsis* (Moulinier-Anzola et al., 2014). TOL6 and TOL9 primarily localize to the plasma membrane and early endosomes, whereas TOL2, TOL3, and TOL5 are found in the cytosol and endosomes (reviewed in Isono, 2020). Their functions may be redundant as multiple combined TOL mutations are required to alter endosomal cargo trafficking (Korbei et al., 2013). Although TOL-binding partners have not yet been identified in plants (a mammalian TOLLIP ortholog could not be identified in plants), it is possible that TOL membrane recruitment is facilitated by binding of their VHS and GAT domains to phosphoinositides as demonstrated in other eukaryotic TOM1 proteins (Blanc et al., 2009; Boal et al., 2015). *Arabidopsis* TOL proteins regulate plant morphogenesis by facilitating the endosomal sorting of a PIN-type auxin carrier protein to the plant lytic vacuoles (Table 2; Korbei et al., 2013). Likewise, TOL5 is required for sorting and degradation of the boron transporter, BOR1 protein (Yoshinari et al., 2018). In agreement with that reported for mammalian TOM1 proteins, TOL2, and TOL6 preferentially bind to Lys63-linked ubiquitin chains via their ubiquitin-binding VHS and GAT domains (Moulinier-Anzola et al., 2020). Specifically, TOL6 has been shown to interact and co-localize at early endosomes with the ESCRT-I component VPS23A (Table 2; Moulinier-Anzola et al., 2020), suggesting that TOL6, and likely the TOL subfamily of proteins, serve as components of the ESCRT-0 complex in plants. TOL proteins have been proposed to negatively regulate the plant immune response as silencing a *Nicotiana benthamiana TOL* gene (an homolog to the human *TOM1-L2*) leads to a reduced growth of bacterial pathogenesis (Conlan et al., 2018). Still, many questions remain to be answered, including why TOL proteins are found in multiple compartments and how, or if, they articulate with other ESCRT members of the protein degradation pathways under physiological and disease conditions.

## Regulation of TOM1 by Phosphoinositides

Phosphoinositides, derived from phosphatidylinositol, are minor but transient phospholipids composed of a phosphorylated myo-inositol ring and two fatty acid chains, which are linked together by a glycerol backbone. Seven biologically relevant phosphoinositides (and the precursor, phosphatidylinositol) can be interconverted by phosphorylation at the 3, 4, and/or 5-OH positions of the inositol ring by specific lipid kinases and phosphatases (Wang et al., 2019), thus, maintaining their spatial and temporal distribution in different subcellular membrane compartments. In addition to their roles in cytoskeletal dynamics, assembly and disassembly of organelle contact sites, mitogenic signaling, and regulation of membrane ion transporters and membrane shape, phosphoinositides are master regulators of endosomal and lysosomal function in coordination with effector proteins (Ebner et al., 2019; Wang et al., 2019).

Accumulated evidence indicates that TOM1 binds phosphoinositides. The homolog of TOM1 in *Dictyostelium*, DdTOM1, was reported to bind phosphoinositides *via* its VHS and GAT domains (Blanc et al., 2009). As DdTOM1 also binds ubiquitin, clathrin, and the ESCRT-I component Tsg101, and since *Dictyostelium* lacks TOLLIP, it is likely that phosphoinositide binding is required for DdTOM1 endosomal membrane targeting (Blanc et al., 2009). In humans, TOM1 GAT weakly binds PtdIns3P at a site overlapping with that for TOLLIP TBD (Figure 3B; Xiao et al., 2015). In the case of the human TOM1 VHS domain, a putative membrane binding positively charged area is located on one side of the protein, whereas a negatively charged patch is oriented on the opposite side (Misra et al., 2000). The positively charged region has been involved in contacts of the TOM1 VHS domain with phosphoinositides, with a preference for PtdIns5P (Boal et al., 2015). PtdIns5P destabilizes the structure of the VHS domain by association to two potential binding sites, one with moderate and another with low affinity for the lipid (Xiong et al., 2019). Multiple mutations on a positively charged patch of the human TOM1 VHS domain (Lys48, Arg52, Lys58, and Lys59), leads to a marked reduction in binding to phosphatidylinositol monophosphates (Boal et al., 2015). However, considering the positively charged nature and the location of these residues in α-helix 3, it is reasonable to argue that these mutations might disrupt the secondary structure of the VHS domain. Therefore, more in-depth structural information is warranted for the identification of the phosphoinositide binding site(s) in the VHS domain. Unrelated proteins, such as the *Trypanosoma brucei* KIFC1 kinesin, a protein involved in the transport of endosomes, associate with membranes containing both acidic lipids and cholesterol through their VHS domains (Lecordier et al., 2020). This cholesterol-binding function allows KIFC1 to modulate cholesterol levels at the plasma membrane, with a direct impact in membrane fluidity (Lecordier et al., 2020). However, it is currently unknown if this cholesterol-binding function is extended to other VHS domains.

## Emerging Physiological and Pathological Functions of TOM1

### Autophagy

Macroautophagy (hereafter autophagy) is a conserved and highly regulated process in eukaryotic cells. Autophagy is initiated under cellular stress conditions, in which double membrane autophagosomes trap large protein aggregates, damaged and aging organelles, and invading pathogens. This cellular material is degraded upon autophagosome fusion with lysosomes. Furthermore, the fusion of multivesicular bodies with autophagosomes, which leads to the formation of amphisomes, provides supplementary protein assemblies (e.g., ESCRT complexes) that are necessary for the subsequent fusion of autophagosomes with lysosomes (Lefebvre et al., 2018). Several proteins, including Myosin VI, TOM1, and an array of ubiquitinated autophagy receptors (NDP52, TAX1BP1, and Optineurin) are involved in amphisome formation (Figure 2C; Tumbarello et al., 2012, 2013; Lamb et al., 2013; Verlhac et al., 2015; Lefebvre et al., 2018; Hu et al., 2019). The motor protein Myosin VI transports cargo along the actin filaments from the plus to the minus end (Hartman and Spudich, 2012). Also, actin filament-dependent spatial organization of endosomes depends upon the presence of both Myosin VI and TOM1 (Masters et al., 2017). During autophagy, TOM1 interacts with the WWY-motif located at the C-terminal CBD domain of Myosin VI (Tumbarello et al., 2012). A high-resolution crystal structure of the complex of Myosin VI’s C-terminal CBD domain with a short stretch (Myosin VI binding motif, MBM; residues 392–463) within the TOM1 C-terminal domain was solved, revealing that the proteins interact through extensive hydrophobic and polar contacts (Hu et al., 2019). TOM1 MBM adopts a long α-helix, which contacts the 4-stranded β-sheet and α-helix 2 of Myosin VI (Figure 3C). Analysis of the structure-based sequence alignment reveals that TOM1 MBM residues are conserved in TOM1-L1 and TOM1-L2 (Figure 1), suggesting that the TOM1 family proteins bind Myosin VI, consequently, supporting the idea of their redundant functions. The RRL motif region of Myosin VI, upstream of the CBD domain, can recognize autophagy receptors and simultaneously interact with TOM1, forming a ternary complex (Figure 2C; Hu et al., 2019). Thus, Myosin VI serves as a bridge to mediate amphisome formation and to promote the maturation of autophagosomes using a multi-binding mechanism (Tumbarello et al., 2012; Hu et al., 2019). The TOM1-binding partner, TOLLIP, may also participate in this process as it localizes to autophagosomes (Lu et al., 2014) and controls the activity of the VPS34, a lipid kinase that regulates the fusion of lysosomes with autophagosomes (Baker et al., 2015). *In vitro* studies showed that polyubiquitin chains can be incorporated to the Myosin VI/TOM1/autophagy receptor (Hu et al., 2019), suggesting that polyubiquitin chains may facilitate amphisome formation (Figure 2C).

### Immune Responses

The immune system must be carefully regulated to allow effective and swift immune responses against pathogens as well as protection of the host against unwarranted responses that could lead to autoimmunity or excessive inflammation. The regulation of many immunological pathways relies on controlled signaling by endosomal compartments associated with cargo sorting and membrane trafficking (Gleeson, 2014). TOM1 was initially proposed to act as a negative regulator of inflammatory signaling induced by IL-1β and TNF-α *via* its VHS domain (Yamakami and Yokosawa, 2004). Later, TOM1, in association with TOLLIP, was reported to sort IL-1R1 at late endosomes for degradation assuring timely resolution of the inflammatory response (Brissoni et al., 2006). TOM1 is also involved in the Toll-like receptors 2/4 (TLR2/4) signaling pathways in cystic fibrosis, an inherited disorder characterized by chronic airway inflammation (Oglesby et al., 2010). Thus, in addition to the regulation of IL-1R1 surface expression, the TOM1-TOLLIP complex may also modulate TLR2/4 recycling by employing a similar mechanism, thereby having an anti-inflammatory role in cystic fibrosis and possibly in other inflammatory diseases.

There are immediate clinical implications of TOM1 in regulation of immunity. Recently, the first disease-causing TOM1 mutation was reported in humans (Keskitalo et al., 2019). A heterozygous *TOM1* p.G307D missense mutation identified in a mother–son pair causes early onset autoimmunity syndrome with arthritis, eczema, enteropathy, and interstitial lung disease. The patients also have a combined immunodeficiency characterized by antibody deficiency, lack of switched memory B cells, dendritic cells, NK cells, and impaired regulatory T cell function. Patient-derived cells display defective autophagy, enhanced apoptosis, and alterations in major immunological signaling pathways including diminished STAT1 and STAT5 tyrosine phosphorylation together with altered ERK1/2 phosphorylation, implying deficient MAPK signaling. The G307D mutation maps at the C-terminus of the GAT domain, a TOLLIP- and ubiquitin-binding domain (Katoh et al., 2004). Indeed, cell-based experiments suggest that TOM1 G307D displays significant reduction in co-localization with both TOLLIP and ubiquitin (Keskitalo et al., 2019), suggesting that the mutation alters the TOLLIP-dependent cargo trafficking function of TOM1.

### Neurodegeneration

Alzheimer’s disease (AD) is an age-related neurodegenerative process that is characterized by symptoms of memory loss and cognitive decline and associated with the formation of senile plaques and neurofibrillary tangles in the hippocampus and cortex. Impairment of protein degradation systems, including the autophagy-lysosome system and the ubiquitin-proteasome system, has been proposed to play a role in the accumulation of these aberrant proteins in AD brains (Uddin et al., 2019). Interestingly, TOM1 as well as its binding partners, TOLLIP and Myosin VI, are found in dystrophic neurites, which occur around senile plaques in the brains of patients with AD (Makioka et al., 2016). At the molecular level, one of the features of AD is the accumulation and deposition of amyloid-β, which promotes neurodegeneration (Chavez-Gutierrez and Szaruga, 2020). Internalization of amyloid-β, mediated by the FcγRIIb2 receptor, is negatively regulated by TOM1, reducing amyloid-β neurotoxicity in AD brains (Gwon et al., 2018). The authors also found that memory impairment in TOM1-expression deficient mice was rescued by replenishing the *TOM1* gene (Gwon et al., 2018), supporting the role of TOM1 in AD-associated memory deficits.

More recently, the association of AD with immune responses has emerged as a result of sustained immune responses in the brain and the development of the AD pathology (Kinney et al., 2018). The endolysosomal system is known to play a role in the downregulation of immune receptors by allowing their traffic for degradation. TOM1, in association with TOLLIP, facilitates the transportation of IL-1β-bound IL-1R1 for degradation (Brissoni et al., 2006), to regulate the extent of the inflammatory response. A recent study shows that levels of TOM1, but not TOLLIP, were markedly decreased in brains of AD patients (Martini et al., 2019). Furthermore, reduction of TOM1 levels was accompanied by an increase of IL-1R1 and IL-1β levels, supporting the role of TOM1 in the downregulation of immune responses. Mice studies have demonstrated amyloid-β deposition and exacerbation of cognitive decay when TOM1 is knocked down, as well as a successful rescue by increasing TOM1 levels (Martini et al., 2019). Taken altogether, TOM1 represents a promising therapeutic target for the restoration of controlled immune responses in aging patients displaying AD pathologies.

### Pathogen-Mediated Hijacking of TOM1

Upon entrance to the host cell, the causal agent of dysentery, *Shigella flexneri*, adheres to and is internalized into epithelial cells through the formation of an entry vacuole (Killackey et al., 2016; Anand et al., 2020). However, in order to escape from the host autophagy machinery, the bacterium is able to breach the endocytic vacuole and move into the cytosol for replication and further invasion (Killackey et al., 2016; Qiu and Cote, 2019). *S. flexneri* employs metabolic strategies to increase cellular levels of PtdIns5P to efficiently survive within the host’s adverse environment (Ramel et al., 2011). PtdIns5P synthesis is mediated by the bacterial enzyme IpgD, a lipid phosphatase that converts plasma membrane phosphatidylinositol 4,5-bisphosphate into PtdIns5P (Niebuhr et al., 2002). IpgD induces a ∼175-fold increase of PtdIns5P levels and triggers the PI3K/Akt pathway through phosphorylation of Akt, a process that also requires the epidermal growth factor receptor (EGFR) (Pendaries et al., 2006; Ramel et al., 2011). Both the PI3K/Akt and EGFR-mediated signaling pathways are involved in cell survival and proliferation, which are beneficial for bacterial survival and replication (Pendaries et al., 2006; Qiu and Cote, 2019). The increased levels of intracellular PtdIns5P at early endosomes also maintain these signaling pathways by blocking EGFR degradation (Ramel et al., 2011; Boal et al., 2015). Upon ligand activation, EGFR is typically degraded through the endosomal trafficking pathway. With PtdIns5P accumulation at signaling endosomes, however, the lipid promotes the activation of ligand-free EGFR and Akt, which are retained for 24–48 h post expression (Pendaries et al., 2006). In addition, TOM1 is recruited to signaling endosomes through PtdIns5P binding (Figure 2D), thereby impairing endosomal maturation and, consequently, inhibiting the degradation of the host cell cargo (Boal et al., 2015). Binding of TOM1 to PtdIns5P is mediated by its N-terminal VHS domain (Boal et al., 2015). As the VHS domain is also a well-known ubiquitin-binding domain, PtdIns5P-dependent relocalization of TOM1 serves as a mechanism for keeping the protein from performing its cargo-sorting function. Production of PtdIns5P by *S. flexneri* is required to dampen the host inflammatory response. For example, an increase of PtdIns5P levels inhibits host ATP secretion (Puhar et al., 2013) and leads to the degradation of the intercellular adhesion molecule-1 (ICAM-1), a glycoprotein that is critical for cell-cell adhesion in response to inflammation (Boal et al., 2016). Curiously, intracellular PtdIns5P levels also increase in many other pathogen infections (Hasegawa et al., 2017), suggesting a common mechanism for pathogen survival in the host cell. Bacterial hijacking of TOM1 can be accompanied by targeting TOM1 protein partners. TOLLIP is also hijacked in *E. coli* CFN1 toxin host entry, which facilitates host cell invasion in a TOM1-dependent manner (Visvikis et al., 2011). Likewise, *Leishmania donovani* hijacks TOLLIP to block IL-1R-mediated innate immunity, facilitating pathogen survival in the host cell (Parmar et al., 2018).

### Cancer

Early studies with mice expressing low levels of TOM1-L2 displayed abnormal immune responses as well as an increased incidence of solid tumors, suggesting that TOM1-L2 might have a role in tumor suppression (Girirajan et al., 2008). Upregulation of another member of the TOM1 family proteins, TOM1-L1, enhances v-erb-b2 avian erythroblastic leukemia viral oncogene homolog 2 gene (ERBB2)-induced invasion of breast cancer cells, in a process that involves TOLLIP (Chevalier et al., 2016). Mechanistically, phosphorylation of TOM1-L1 at Ser321 within the GAT domain increases the affinity of the protein for TOLLIP, which in turn, facilitates the membrane localization of the membrane-type 1 matrix metalloprotease. Activation of this enzyme exerts invadopodia tumor cell activity by degrading the extracellular matrix and therefore facilitating tumor cell dissemination (Chevalier et al., 2016). More recently, using genome-wide association studies, it was discovered that the *TOM1* gene represents a multiple myeloma risk allele (reviewed in Pertesi et al., 2020) although the mechanistic evidence of such association remains unknown.

## Conclusion and Perspectives

Cargo sorting at the endosomal compartments is driven by the ESCRT machinery. Intriguingly, together with TOM1, many other ubiquitin-binding modular proteins have been grouped within the ESCRT-0 family in mammals. Some specifically recognize Lys48-linked ubiquitin moieties, whereas others like TOM1 and TOLLIP prefer Lys63-linked over Lys-48-linked polyubiquitin chains covalently attached to protein receptors (Nathan et al., 2013). These differences perhaps can explain why a variety of ESCRT-0 are expressed in mammals. A growing body of evidence strongly supports that TOM1 actively participates in the trafficking of cargo at the surface of endosomal compartments in coordination with other ESCRT proteins. Many TOM1 protein partners have been reported in mammals, with TOLLIP taking the lead as the major protein partner for cargo sorting. Still, how TOM1 can choose one protein partner over another remains unknown. TOM1 displays two ubiquitin-binding domains, the VHS and GAT domains (Misra et al., 2000; Akutsu et al., 2005; Xiao et al., 2016). There is still no evidence whether these two TOM1 domains can bind to the same cargo or if each associate to different cargo. Also, it is not known whether the VHS and GAT domains preferentially bind a specific ubiquitin topology in either TOM1 free state or when bound to other ESCRT-0 proteins. In plants, the role of TOL (TOM1 homolog in plants) seems to be simpler, as no protein partners for TOL proteins have been yet reported. TOLs, components of the plant ESCRT machinery, function as a sorting tool for cargo degradation in the vacuole pathway (Moulinier-Anzola et al., 2020). TOLs interact with ubiquitinated cargo *via* their VHS and GAT domains, associate with phosphoinositides for membrane targeting at early endosomes, late endosomes, the Golgi apparatus, and the plasma membrane, and likely interface with ESCRT-I proteins (Korbei et al., 2013; Mosesso et al., 2019; Moulinier-Anzola et al., 2020). Multiple TOL isoforms with overlapping functions have been identified in plants, which may be required to quickly remove membrane cargo under environmental changes that require rapid adaptation.

Recent novel functions of TOM1 in mammals were underscored. One of the most important was its role in mediating the fusion of endosomes with autophagosomes in a mechanism that depends upon the action of Myosin VI, which serves as a bridge for TOM1 proximity with the autophagic receptors NDP52, TAX1BP1, and Optineurin (Hu et al., 2019). This new role of TOM1 in autophagy can potentially be powered by the PtdIns3P-binding proteins TOLLIP, Endofin, or Hrs, which may serve as a bridge for TOM1 membrane recruitment to these compartments. PtdIns5P has been shown to stimulate autophagy under physiological conditions (Vicinanza et al., 2015). Thus, as a PtdIns5P-binding protein, TOM1 can be directly recruited to endosomal or autophagosomal membranes through this phosphoinositide interaction without the presence of another phosphoinositide-binding protein. Importantly, the mechanism which triggers TOM1 to commit to either cargo trafficking or amphisome formation remains elusive.

As TOM1 family proteins have been involved in processes that primarily pertain to membrane dynamics, recent reports that associate them with aberrant protein trafficking (i.e., Alzheimer’s disease, bacterial hijacking, cancer) are not surprising. Furthermore, a mutation in TOM1 has been described in patients with severe immunodysregulation syndrome pointing out a significant role of TOM1 in controlling excess inflammation as well as development of normal immunity. Accordingly, a better understanding of the function and regulation of TOM1 proteins is warranted to develop novel therapeutics for cancer as well as, infectious, autoimmune, and neurological diseases.

## Author Contributions

All authors listed have made a substantial, direct and intellectual contribution to the work, and approved it for publication.

## Conflict of Interest

The authors declare that the research was conducted in the absence of any commercial or financial relationships that could be construed as a potential conflict of interest.

## Footnotes

1 https://www.proteinatlas.org
